# Relations Between the Use of Electronic Health and the Use of General Practitioner and Somatic Specialist Visits in Patients With Type 1 Diabetes: Cross-Sectional Study

**DOI:** 10.2196/11322

**Published:** 2018-11-07

**Authors:** Anne Helen Hansen, Jan Broz, Tor Claudi, Eirik Årsand

**Affiliations:** 1 Centre for Quality Improvement and Development University Hospital of North Norway Tromsø Norway; 2 Department of Community Medicine Faculty of Health Sciences UiT - The Arctic University of Norway Tromsø Norway; 3 Department of Internal Medicine Second Faculty of Medicine Charles University Prague Czech Republic; 4 Medical Department Nordland Hospital Bodø Norway; 5 Norwegian Centre for E-health Research University Hospital of North Norway Tromsø Norway; 6 Department of Clinical Medicine UiT - The Arctic University of Norway Tromsø Norway

**Keywords:** eHealth, internet, health care utilization, general practitioners, specialist, cross-sectional study, diabetes mellitus, type 1, Norway

## Abstract

**Background:**

The prevalence of diabetes and the use of electronic health (eHealth) are increasing. People with diabetes need frequent monitoring and follow-up of health parameters, and eHealth services can be of great value. However, little is known about the association between the use of eHealth and provider-based health care services among people with diabetes.

**Objective:**

The objective of this study was to investigate the use of 4 different eHealth platforms (apps, search engines, video services, and social media sites) and associations with the use of provider-based health care visits among people diagnosed with type 1 diabetes mellitus (T1DM).

**Methods:**

We used email survey data collected from 1250 members of the Norwegian Diabetes Association (aged 18 to 89 years) in 2018. Eligible for analyses were the 523 respondents with T1DM. Using descriptive statistics, we estimated the use of eHealth and the use of general practitioners (GPs) and somatic specialist outpatient services. By logistic regressions, we studied the associations between the use of these provider-based health services and the use of eHealth, adjusted for gender, age, education, and self-rated health.

**Results:**

Of the sample of 523 people with T1DM, 90.7% (441/486) had visited a GP once or more, and 61.0% (289/474) had visited specialist services during the previous year. Internet search engines (such as Google) were used for health purposes sometimes or often by 84.0% (431/513), apps by 55.4% (285/514), social media (such as Facebook) by 45.2% (232/513), and video services (such as YouTube) by 23.3% (118/506). Participants aged from 18 to 39 years used all forms of eHealth more than people aged 40 years and older, with the exception of social media. The use of search engines was positively associated with the use of somatic specialist services (odds ratio 2.43, 95% CI 1.33-4.45). GP visits were not associated with any kind of eHealth use.

**Conclusions:**

eHealth services are now widely used for health support and health information by people with T1DM, primarily in the form of search engines but often in the form of apps and social media as well. We found a positive association between the use of search engines and specialist visits and that people with T1DM are frequent users of eHealth, GPs, and specialist services. We found no evidence that eHealth reduces the use of provider-based health care; these services seem to be additional rather than alternative. Future research should focus on how health care services can meet and adapt to the high prevalence of eHealth use. Our results also indicate that many patients with T1DM do not visit specialist clinics once a year as recommended. This raises questions about collaboration in health care services and needs to be followed up in future research.

## Introduction

### Background

Internet-based health information, sensors, apps, and other solutions for self-management, as well as new treatment strategies have developed rapidly in recent years, becoming an important support for both patients and health services. Of particular interest in this regard are patients with chronic diseases, such as diabetes, who are in need of frequent monitoring and follow-up of health parameters.

### Increasing Prevalence of Diabetes Mellitus

The prevalence of diabetes is increasing worldwide. Estimates of 415 million cases in 2015 (age group 20-79 years) are expected to rise to 642 million in 2040 [1]. Global prevalence in adults is estimated at 8.8% [1] and the Norwegian prevalence at 4.7% [2]. Around 245,000 persons are diagnosed with diabetes in Norway, of whom around 28,000 have type 1 diabetes mellitus (T1DM) [2]. Costs attributable to diabetes represent around 1.4% of the total Norwegian expenditure on health care [3]. Diabetes is a considerable burden on patients in terms of morbidity and mortality [4]. Most patients do not reach the combined national treatment targets for prevention of complications [5-7].

### Increasing Use of Electronic Health Services

The World Health Organization states that “eHealth is the use of information and communication technologies (ICT) for health” [8]. The use of electronic health (eHealth) has increased over the past decades. Back in 2005, 44% of the general population of 7 European countries reported using the internet for health purposes [9,10], increasing to 52.2% by 2007 [11]. Consistent with European trends, in Poland, 66.7% used the internet for health purposes in 2012 [12]. Around 75% to 80% of internet users in the United States and Europe conduct health-related searches [9,13]. Most Norwegian households (97%) had internet access in 2015 [9-11,14], and 78% of the population aged 15 years and older have reported using the internet for health purposes [15]. In the Czech Republic, more than 25% of insulin-treated patients visited a professional diabetes internet portal in the period between 2009 and 2013 [16]. However, eHealth use among people with T1DM in Norway has yet to be explored.

### Unclear Relations Between the Use of Electronic Health and Provider-Based Health Services

Andreassen et al found that the use of eHealth in a general population was positively associated with general practitioner (GP) visits (yes or no) [10], whereas others have reported no or inverse associations with the *frequency* of regular provider visits [17,18]. A German study found that frequent users of health services were 73% more likely to seek health information on the internet compared with nonusers [12]. Research on the associations between the use of eHealth and provider-based health care is scarce, both in general populations and for populations with specific diseases [19,20].

### Norwegian Health Care Services

The Norwegian health care system is based on universal insurance. Primary health care is run by the municipalities. All residents are provided a regular GP according to the patient list system. Specialist outpatient services are operated by regional and local health enterprises owned by the national government, consisting of public and private somatic and psychiatric specialist services. Access to specialist care is usually achieved by referral from the regular GP (the gatekeeper role). However, persons with T1DM are recommended to make at least one annual visit to specialist health services [21] and are most often invited directly for annual checks. GP and specialist visits for adults have a small co-payment, with a total maximum limit of 2258 Norwegian Kroner (around US $280) within a year (2018).

### Planning for Future Electronic Health and Provider-Based Health Care Services

The use of eHealth is an area of continuous and rapid development, which varies between regions, countries, diagnostic groups, health care services, and health care systems. Hence, research from different settings is important to achieve an overall epidemiological view. A comprehensive understanding of the influence of eHealth on health care utilization in patients with T1DM is thus important for patients, health care providers, administrators, policy makers, and society to enable evidence-based planning for future eHealth and provider-based health care services.

### Aim

The aim of this study was to investigate which eHealth services are used among people with T1DM and whether the use of eHealth is associated with the use of primary and specialist health care services. Specifically, we tested whether the use of apps, search engines (such as Google), video services (such as YouTube), and social media (such as Facebook) was associated with the use of GPs and somatic outpatient specialist services.

## Methods

### Data

For this cross-sectional study, we used email survey data obtained in January and February 2018 from members of the Norwegian Diabetes Association. On December 31, 2017, the organization had 33,908 members—53% women and 47% men. Around 30% of the members have T1DM [22]. The Norwegian Centre for Research Data (NSD) Web survey distributed the invitations to a randomly selected sample of 5971 individuals (about 18% of all members).

**Figure 1:**
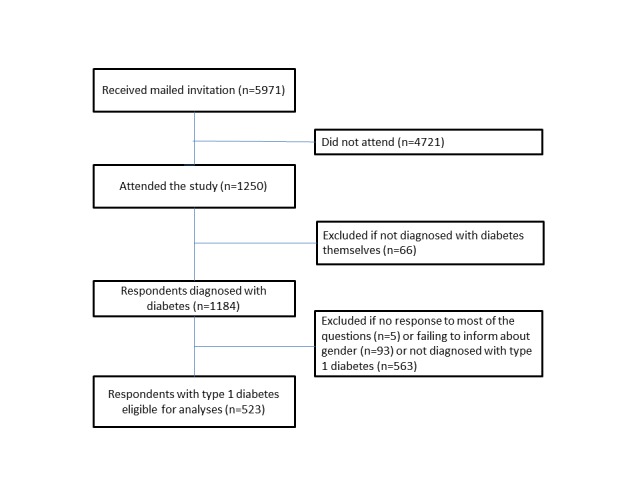
Flowchart of study population.

Initially, as described in our protocol paper [23], we planned to use data from the seventh Tromsø study conducted in 2015-2016, where we participated in the planning. Comprehensive population-based health surveys have been conducted in Tromsø since 1974. In the seventh study, all residents aged 40 years and older (around 33,000 persons) were invited, and questions about the use of eHealth were included for the first time. However, after data collection, the Tromsø study was not able to give us access. Consequently, we had to change our data collection plans. We developed a tailored questionnaire based on the specific objectives of our study [23], using relevant questions from other published surveys on health care utilization and health information seeking [24,25].

Information about the study purpose and what participation would entail was distributed together with the invitation. The questionnaire (Multimedia Appendix 1) included questions about demographic and socioeconomic characteristics, health status including diabetes-specific questions about duration, severity and treatment, and use of and experiences with eHealth and health care services. Before data collection, the questionnaire was reviewed and tested several times by 2 persons diagnosed with diabetes and by experts from our research group (EÅ and AHH). Nonrespondents were given 1 reminder submitted by email 15 days after the first request.

### Participants

It was not possible for the same respondent to fill in the questionnaire more than once. Starting from 1250 participants, we first excluded those who did not suffer from diabetes themselves (n=66). This group consisted of 61 family members, 4 health personnel (2 overlapping), and 3 others. We also excluded participants who failed to respond to most of the questions (n=5) and those who did not give information about gender (n=93). Finally, as we had decided to investigate T1DM in this part of the study, participants with type 2 diabetes mellitus (T2DM) and other diabetes types were excluded. The sample finally consisted of 523 respondents (Figure 1).

### Variables

The dependent variables were use of GPs and outpatient specialist services during the previous 12 months. Specialist services use refers to any somatic specialist clinic visit, regardless of clinical issue (not only endocrinologists/diabetologists). For GP services, 2 dichotomous outcome variables were applied, 1 for use or no use and 1 for less frequent use (0 to 2 visits) or more frequent use (3 visits or more). The distinction between more and less frequent use was the 50th percentile, a cutoff point that has been used in previous research [26]. For outpatient specialist visits, the 50th percentile was set at 1, making a frequency variable redundant.

Respondents were asked about their use of eHealth in the same period. eHealth was subdivided into apps for mobile phone or tablet computer, search engines (such as Google), social media (such as Facebook), and video services (such as YouTube). eHealth variables were dichotomized by merging the original 4 answering options into “never or once” and “sometimes or often.”

The use of eHealth (apps, search engines, social media, and video services) was the key independent variable. Adjustment independent variables were gender, age, education, and self-rated health. We grouped age in 20-year intervals. The 4 education categories were labeled as follows: low (primary/part of secondary school), middle (completed secondary school), high (college/university<4 years), and highest (college/university 4 years or more). Response options for self-rated health were excellent, good, fair, bad, and very bad. The bad and very bad categories were merged because of low numbers in the very bad category (4 respondents).

### Analyses

Data were analyzed by means of descriptive statistics and logistic regressions. Correlations were tested with Spearman as well as Pearson correlation coefficients. We constructed 1 multivariable regression model for each of the dependent variables. The independent variables (apps, search engines, social media, video services, gender, age, education, and self-rated health) were introduced collectively into the models.

Due to a relatively low response rate, we compared respondents who did not respond initially but eventually consented with early respondents, assuming that late respondents were more similar to nonrespondents [27]. This was done by descriptive statistics (stratification), and by subsequently introducing the response time variable into the regression models.

We used 95% CIs throughout the study. All analyses were accomplished using Stata, version 14.2.

### Ethics

This project has been presented to the Regional Committee for Medical and Health Research Ethics (REK), which found that an application was not required according to the Norwegian Health Research Act (2015/1779/REK nord). The study has been approved by the data protection officer (Personvernombudet) at the University Hospital of North-Norway (ref 2017/6579). The data bureau NSD received no other information about the participants than the email addresses.

## Results

### Participation

In total, 1250 persons aged 18 to 89 years participated, constituting a minimum response rate of 20.93% (1250/5971) (Figure 1). However, we experienced more than 400 bounce backs from servers unable to deliver the invitation. Consequently, the real response rate is assumed higher. Eligible for analysis in our study were the 523 persons who reported having T1DM (Figure 1).

### Sample Characteristics

Mean age was 47.0 years, 48.9 years for men and 45.3 years for women. Median age was 48 years. Mean disease duration was 23.2 years (median 22 years). Most participants (75.7%) had suffered from diabetes for 10 years or more. The largest groups consisted of women (281/523, 53.7%), persons aged 40-59 years (223/523, 42.6%), married/cohabitants (338/380, 89.0%), full-time or part-time employed persons (308/481, 64.0%), persons with high education (152/480, 31.7%), high household income (238/467, 51.0%), good self-rated health (269/521, 51.6%), and good self-rated regulation of diabetes (292/520, 56.2%). Among the late respondents (those who responded after the reminder), older people (60 years and older) represented a larger proportion (57/180, 31.7% compared with 65/343, 19.0% among the early respondents Table 1).

### The Use of Electronic Health and Provider-Based Health Care Services

During the previous year, 90.7% (441/486) visited a GP once or more, and 61.0% (289/474) visited somatic outpatient services (Table 2). Overall, 87.0% (447/514) used eHealth in one or more forms. Search engines were used sometimes or often by 84.0% (431/513), apps were used by 55.4% (285/514), social media were used by 45.2% (232/513), and video services were used by 23.3% (118/506) of participants (Table 2). People aged 40 years and older used all health care services more and all forms of eHealth less than younger people, with the exception of social media (Table 2).

### Relations Between the Use of Provider-Based Health Care Services and Electronic Health

GP visits (yes or no, and frequency) were not associated with any type of eHealth use. The use of somatic outpatient services was positively associated with the use of search engines (odds ratio [OR] 2.43, CI 1.33-4.45; Table 3). The use of apps, social media, or video services was not associated with the use of health care services (Table 3).

The probability of 1 or more GP visits during the previous year increased with poorer health. People in good and fair self-rated health were more likely to visit the GP compared with those in excellent health (OR 2.20, CI 1.04-4.68 and OR 6.73, CI 1.78-25.49, respectively). All the 42 individuals reporting bad/very bad health had visited their GP during the previous year. As there were 0 observations in the nonvisiting group; OR was not calculated (Table 3).

The probability of more frequent GP visits also increased with poorer health and was almost 7 times higher in the bad/very bad health group compared with those in excellent health (OR 6.77, CI 2.63-17.45).

People in bad/very bad health were more than twice as likely to visit specialist services compared with those in excellent health (OR 2.51, CI 1.07-5.88).

**Table 1:** Percentage of sample characteristics.

Variables		Total sample, n	Early respondents, n	Late respondents, n	18-39 years, n	40-59 years, n	60 years and older, n
**Gender**		523	343	180	178	223	122
	Female	53.7	52.2	56.7	59.5	52.0	48.4
	Male	46.3	47.8	43.3	40.5	48.0	51.6
**Age (years)**		523	343	180	178	223	122
	18-39	34.0	37.0	28.3	N/A^a^	N/A	N/A
	40-59	42.6	44.0	40.0	N/A	N/A	N/A
	60 and above	23.4	19.0	31.7	N/A	N/A	N/A
**Marital status**		380	254	126	129	163	88
	Single	11.0	13.8	5.6	27.1	4.3	0.0
	Married/cohabitant	89.0	86.2	94.4	72.9	95.7	100.0
**Main daily activity**		481	320	161	155	210	116
	Working^b^	64.0	67.5	57.1	66.4	81.9	28.5
	Pensioner old age	13.5	10.6	19.3	0.0	0.0	56.0
	Pensioner disability	11.0	9.4	14.3	5.8	14.3	12.1
	Pupil/student	7.3	8.1	5.6	22.6	0.0	0.0
	Other	4.2	4.4	3.7	5.2	3.8	3.4
**Education^c^**		480	319	161	155	210	115
	Low	8.1	6.3	11.8	5.2	7.6	13.0
	Middle	29.0	27.9	31.1	32.9	26.7	27.8
	High	31.7	33.8	27.3	29.7	32.9	32.2
	Highest	31.2	32.0	29.8	32.2	32.8	27.0
**Household income^d^**		467	309	158	153	207	107
	Low	14.1	13.3	15.8	24.2	7.7	12.2
	Middle	34.9	33.3	38.0	37.3	26.1	48.6
	High	51.0	53.4	46.2	38.5	66.2	32.2
**Duration of diabetes**		522	343	179	178	222	122
	<10 years	24.3	23.3	26.2	36.0	17.6	19.7
	10-19 years	20.5	20.7	20.1	28.6	17.6	13.9
	20-29 years	19.4	20.7	16.8	27.5	17.6	10.7
	30 years and above	35.8	35.3	36.9	7.9	47.2	55.7
**Self-rated regulation of diabetes**		520	341	179	178	222	120
	Excellent	19.4	17.0	24.0	19.1	17.6	23.3
	Good	56.2	57.2	54.2	50.0	57.7	62.5
	Fair	19.8	20.5	18.4	23.6	20.7	12.5
	Bad/very bad	4.6	5.3	3.4	7.3	4.0	1.7
**Self-rated health**		521	342	179	178	222	121
	Excellent	17.9	15.5	22.4	14.6	19.4	19.8
	Good	51.6	53.2	48.6	59.6	47.3	47.9
	Fair	21.7	21.9	21.2	15.7	23.4	27.3
	Bad/very bad	8.8	9.4	7.8	10.1	9.9	5.0

^a^N/A: not applicable.

^b^Full-time or part-time.

^c^Low (primary/part of secondary school), middle (completed secondary school), high (college/university <4 years), and highest (college/university 4 years or more).

^d^Low (Norwegian Kroner [NOK] <350,000), middle (NOK 351,000-750,000), high (NOK >750,000).

**Table 2:** Proportion using provider-based health care services and 4 types of electronic health (eHealth) services during the previous 12 months.

Variables		Total T1DM^a^ sample, n (%)	Early respondents, n (%)	Late respondents, n (%)	18-39 years, n (%)	40-59 years, n (%)	60 years and above, n (%)
**Use of health care services once or more**							
	GP^b^	441 (90.7)	293 (90.4)	148 (91.2)	132 (84.1)	198 (93.8)	111 (94.1)
	Somatic outpatient specialist	289 (61.0)	182 (58.2)	107 (66.5)	82 (53.3)	140 (67.3)	67 (59.8)
**Use of eHealth sometimes or often**							
	Apps	285 (55.4)	194 (57.6)	91 (51.4)	108 (62.4)	121 (55.3)	56 (45.9)
	Search engines	431 (84.0)	293 (86.9)	138 (78.4)	159 (91.9)	184 (84.4)	88 (72.1)
	Social media	232 (45.2)	154 (45.8)	78 (44.1)	78 (45.1)	108 (49.5)	46 (37.7)
	Video services	118 (23.3)	80 (24.1)	38 (21.8)	45 (26.5)	51 (23.5)	22 (18.5)

^a^T1DM: type 1 diabetes mellitus.

^b^GP: general practitioner.

Among people aged 60 years and older, the probability of visiting a GP was more than 5 times higher than in the youngest (18 to 39 years) group (OR 5.25, CI 1.82-15.14). The probability of frequent visits was twice as high among people aged 60 years and older (OR 1.98, CI 1.15-3.40). The probability of visiting specialist services was significantly higher among people aged from 40 to 59 years, compared with the youngest group (OR 1.97, CI 1.25-3.11), but not significantly higher among those aged 60 years and older (Table 3).

The group with high education was more likely to visit somatic outpatient services compared with the low education group (OR 2.97, CI 1.36-6.51). GP visits were not associated with educational level. Gender was not associated with the use of health care services (Table 3). Goodness of fit was tested by Hosmer/Lemeshow goodness-of-fit test. All tests showed nonsignificant *P* values, indicating acceptable fit for all models.

All findings regarding GP visits persisted after introducing the response time variable into the regression models. The probability of using somatic outpatient services was increased among the late respondents compared with the early respondents (OR 1.84, CI 1.18-2.86). The positive association between the use of somatic outpatient services was increased among those in fair health (OR 2.04, CI 1.07-3.91) and among those in bad/very bad health (OR 2.74, CI 1.16-6.45). Otherwise, the introduction of the response time variable did not alter the results.

There were no strong correlations (defined as Spearman rho >.5) between the independent variables in any of the models. A similar result was found using Pearson correlation test.

**Table 3:** Probability of using general practitioners (GPs) and somatic outpatient services during the previous year in a population with diabetes type 1 (multivariable logistic regressions).

Variables		GP visits (yes/no; n=432), OR^a^(95% CI)	GP visits (0 to 2 vs 3 visits or more; n=474), OR (95% CI)	Somatic outpatient visits (yes/no; n=468), OR (95% CI)
Apps^b^		1.29 (0.62-2.70)	1.21 (0.77-1.88)	0.65 (0.41-1.02)
Search engines^b^		0.92 (0.33-2.52)	1.10 (0.60-2.02)	*2.43*^*c*^ (*1.33-4.45*)
Social media^b^		1.33 (0.59-3.01)	1.38 (0.88-2.17)	1.15 (0.73-1.82)
Video services^b^		1.73 (0.64-4.70)	0.94 (0.56-1.56)	1.06 (0.64-1.76)
Gender^d^		0.64 (0.32-1.29)	0.77 (0.51-1.15)	0.99 (0.66-1.49)
**Age, in years**				
	18-39^e^	1.00	1.00	1.00
	40-59	*3.50* (*1.63-7.52*)	*1.74* (*1.10-2.75*)	*1.97* (*1.25-3.11*)
	60 and above	*5.25* (*1.82-15.14*)	*1.98* (*1.15-3.40*)	1.63 (0.96-2.79)
**Education^f^**				
	Low^e^	1.00	1.00	1.00
	Middle	2.48 (0.66-9.26)	1.03 (0.46-2.33)	1.69 (0.78-3.66)
	High	2.66 (0.70-10.11)	1.17 (0.52-2.64)	*2.97* (*1.36-6.51*)
	Highest	1.56 (0.44-5.59)	0.70 (0.31-1.58)	2.16 (0.98-4.74)
**Self-rated health^f^**				
	Excellent^f^	1.00	1.00	1.00
	Good	*2.20* (*1.04-4.68*)	1.35 (0.80-2.28)	1.43 (0.84-2.42)
	Fair	*6.73* (*1.78-25.49*)	*4.15* (*2.15-8.01*)	1.86 (0.98-3.52)
	Bad/very bad^g^	N/A^h^	*6.77* (*2.63-17.45*)	*2.51* (*1.07-5.88*)

^a^OR: odds ratio.

^b^Apps/search engines/social media/video services in 2 groups: 1=never or once, 2=sometimes or often.

^c^Statistically significant findings are marked in italics.

^d^Gender: 1=women, 2=men.

^e^Reference groups.

^f^Education: low (primary/part of secondary school), middle (high school), high (college/university <4 years), highest (college/university 4 years or more).

^g^Odds ratio was not calculated for general practitioner visits (yes/no) because of zero (0) observations in the nonvisiting group.

^h^N/A: not applicable.

## Discussion

### Principal Findings

We found that 90.7% (441/486) of study participants visited a GP once or more during the previous year, and 61.0% (289/474) participants visited somatic outpatient services. Search engines were used sometimes or often by 84.0% (431/513), apps by 55.4% (285/514), social media by 45.2% (232/513), and video services by 23.3% (118/506) of the participants. Participants aged 18 to 39 years used the investigated forms of eHealth more than people aged 40 years and above, with the exception of social media. GP visits were not associated with the use of eHealth, whereas visits to specialist services were positively associated with the use of search engines. Poorer self-rated health and higher age were associated with increased use of GPs and specialist services, whereas higher education was associated with increased use of specialist services. Gender, social media use, and video services use were not associated with the use of health services.

### Low Specialist and High General Practitioner Visit Rates

We were surprised that only 61.0% (289/474) reported 1 or more visits to any somatic specialist service during the previous 12 months. This is a higher rate than reported for the general population [28] but still remarkably low as at least one annual checkup visit is recommended for people diagnosed with T1DM [21]. In Salten, Norway, around 80% of insulin-treated patients reported visits to specialist services regarding their diabetes during 2014 (unpublished data from the Rosa4 study, communicated by TCL). We have not found other studies reporting specialist checkup rates for patients with T1DM in Norway; however, it has been suggested that many older patients are monitored by their GP [7]. Our finding that people aged 60 years and older less likely visited specialists compared with people aged 40 to 59 years (Table 3) supports this view. However, it is important to note that low specialist visit rates apply to all age groups, and in particular to younger ages (18 to 39 years, Table 2).

On the other hand, a GP visit rate of 90.7% (441/486) is high compared with around 80% in the general population [25,28]. Patients with T1DM might see their GP for a variety of health problems that are connected, directly or indirectly, to their diabetes. A few GPs are specialized in diabetes care and might partly provide a substitute for specialist clinic checkup. This might explain some of the low specialist visit rate, but it is unlikely to explain all of it. The notion that people with T1DM are followed up by annual checkups in specialist services thus needs to be questioned or at least nuanced. It may be problematic if the GP and the specialist both believe that the other is performing the checkup of their patients, with the risk of dropouts. Our finding raises questions about the collaboration between GPs, specialist health services, and patients and needs to be followed up in further research.

### Extensive Use of Electronic Health

Our finding that people with T1DM are heavy users of all 4 forms of eHealth is not surprising. Among people aged 40 years and older, the different types of eHealth were used from around twice (search engines) to 5, 6, or 7 times (apps, video services, and social media) more than reported for the general population in the Tromsø Study [25]. However, it should be noted that our data were collected 2 to 3 years later than the Tromsø Study. Considering the rapid development in this field, some of the differences might be due to changing of trends over time. Nevertheless, reports are quite consistent that people with chronic conditions or poorer self-rated health are more likely to use eHealth than the general population [29-33]. This conforms with the illness behavior model [34], indicating that people in poor health are more likely to seek Web-based disease-related information, where an obvious prerequisite is access [35]. Concerns about one’s own disease or poor health will naturally lead to demand for relevant information. This extensive use may reinforce the notion that eHealth and provider-based health care are additional rather than alternative services at present, possibly interacting in a reciprocal way [33,36] and that this applies at least as much to people with T1DM as to the general population.

### The Age Divide

This study confirms that younger people use the internet for health purposes more than older people, particularly apps and search engines. Previous research is consistent regarding this age divide for general populations, elderly populations, and populations with chronic disease [10,11,15,17,33,37-41]. Findings by Tarver et al may indicate that age differences among internet users decreased in the period from 2003 to 2013, although this finding was not statistically significant [41]. This possible trend may amplify when cohorts exposed to digital technology from childhood get older, and the present inverse association between age and eHealth use might not be sustained to the same extent in the future. Elderly people are a rapidly growing age group in Europe and a fast-growing group of eHealth users [12,42].

### Positive Association Between Specialist Visits and the Use of Search Engines

GP visits were not associated with any kind of eHealth use, whereas specialist visits were positively associated with the use of search engines. Back in 2008, Lee suggested that internet use for health information increased contact with health professionals [36]. In line with this, 2 Asian studies recently found that internet use was significantly associated with more outpatient clinic visits [29,43]. Medlock’s study of elderly people in the Netherlands, however, reported that use of health professionals was not associated with internet use [24]. None of these studies can be directly compared with this study, as study methodology, health care systems, and cultures differ substantially, and the other studies were not performed in disease-specific populations. The specific finding of an association between specialist visits and the use of search engines among people with T1DM might point to a need for additional information concerning specialist visits, which may be greater than the need connected to GP visits. We know that eHealth might be used *before* the visit to seek information or to decide about the need to see a doctor and *after* the visit for additional information [31,33,44]. The GP-patient relationship may have a longer duration, and GP visits may be more frequent than specialist visits. Thus, a closer relationship with continuity combined with room for questions and discussions may develop [45]. In Norwegian specialist care, the patient will not necessarily see the same physician from 1 visit to the next, and the internet may be of great value as a source of supplemental information.

### Self-Rated Health, Education, and Late Respondents

People in poorer self-rated health used the surveyed provider-based health services significantly more than people in better health. This adds to solid documentation in previous research, both for disease-specific and general populations [26,29].

The probability of visiting somatic specialist services was higher among those with higher education, even if health is usually worse in lower socioeconomic groups. Our finding is consistent with previous findings for the general Norwegian population [26].

The increased probability of using somatic outpatient services among the late respondents in our study is most likely because of the higher age in this group and the consequences related to increased health care needs. Otherwise, our investigation of early versus late respondents did not alter the results.

### Limitations

The main limitation of this study is the low participation rate, one of the indicators of study representativeness [46]. However, response rate must not be confused with response quality [27]. More important is the assessment of the possible influence of nonparticipation on exposure, outcome, or the relation of interest [47]. In our study, older people dominated among the late respondents compared with the early respondents. Assuming that late respondents are more similar to nonrespondents, younger individuals may be overrepresented in our study.

The distribution of the questionnaire to people with email addresses excluded those who do not use the internet or do not have an email address. The distribution of functioning email addresses might have been skewed, for instance, toward younger members. However, as 97% of Norwegian households have internet access, 90% of Norwegians use the internet every day, and around 91% use email [42], we do not think this has affected our results significantly. Nevertheless, some of those invited might not use email regularly, and some may use mobile phones more than computers. In both cases, there might be barriers to filling out a large questionnaire. In Norway, younger people use email less and mobile phones more than middle-aged and older people [42]. In addition, participation in surveys is generally lower among younger people [48]. These factors might contribute to balance a possible overrepresentation of younger people in this study, and consequently add to its generalizability.

It is well known that women, healthier persons, higher socioeconomic groups, and middle-aged people are more likely to participate in surveys [47,48]. This suggests that women, people aged from 40 to 80 years, people in better health, and higher socioeconomic groups might be overrepresented in our study, thus tending to level out a possible skewness in the opposite directions.

Furthermore, we presume that people with interest in eHealth might be overrepresented, as interest in the topic studied has shown to increase responses [49]. If this is the case, our rates of eHealth use might be higher than the true rates. However, this point applies to most other eHealth studies as well.

In questionnaire data, there is always a potential for recall bias, particularly regarding minor events and distant past, usually leading to underreporting [50]. In addition, the validity of self-reported data of health care utilization may be questioned, although agreement between self-reported and registered health care use is generally high [51]. The cross-sectional study design implies that no causal relationships can be established. Furthermore, we cannot exclude the possibility of unmeasured confounders of the reported associations.

Overall, we conclude that younger individuals might be overrepresented in this study. It is not possible to judge the magnitude of a possible bias, as different factors might pull the tendency in different directions or level each other out. The low response rate is in itself not an indication of low representativeness, as nonresponse bias may be a problem even if response rates are high [52]. Moreover, our results seem reasonable and not contradictory to prior research where such research is available. We suggest that bias poses a limited threat to our study’s validity. Nevertheless, generalization must be approached with caution.

### Strengths

One strength of this study is the focus on an area that has been scarcely investigated. Another strength is the fact that we were able to design a more detailed questionnaire specifically tailored to people with diabetes. This enabled us to distinguish between T1DM and T2DM. Further, we were able to recruit participants from all of Norway, not only from Tromsø municipality, as planned in the protocol using data from the Tromsø Study. This study included individuals from 18 years of age, whereas participants in the Tromsø Study were 40 years and older. Moreover, we were able to analyze data shortly after they were collected, which we consider of great importance in the rapidly developing field of eHealth. Finally, yet importantly, the collection of data in cooperation with the Norwegian Diabetes Association enabled us to develop excellent user participation with a large and important group of health care users.

### Future Plans

We plan to extend this study by investigating how eHealth use and use of other health care services, such as emergency departments and hospitalizations, might be related. Furthermore, we will make efforts to contribute to a deeper understanding of possible causal relationships regarding the use of eHealth and provider-based health care services. In further studies, this will be applied to populations with T2DM as well as T1DM.

### Conclusions

We found that the eHealth services are widely used for health information by people with T1DM, primarily in the form of search engines, but often in the form of apps and social media as well. Our study suggests a positive association between the use of search engines and specialist visits and that people with T1DM are frequent users of eHealth, GPs, and specialist services. We found no evidence that eHealth reduces the use of provider-based health care, suggesting that these services are additional rather than alternative in today’s health care. For future research, it would be interesting to investigate how eHealth services may replace some of today’s face-to-face consultations. Moreover, our results indicate that many patients with T1DM do not visit specialist clinics once a year as recommended. This raises questions about the collaboration between GPs, specialist services, and patients and needs to be followed up in future research.

## Appendix

Multimedia Appendix 1 Questionnaire.

## Abbreviations

eHealth: electronic health
GP: general practitioner
NSD: Norwegian Centre for Research Data
OR: odds ratio
REK: Regional Committee for Medical and Health Research Ethics
T1DM: type 1 diabetes mellitus
T2DM: type 2 diabetes mellitus
